# Brown and Beige Adipose Tissue and Aging

**DOI:** 10.3389/fendo.2019.00368

**Published:** 2019-06-20

**Authors:** Elena Zoico, Sofia Rubele, Annamaria De Caro, Nicole Nori, Gloria Mazzali, Francesco Fantin, Andrea Rossi, Mauro Zamboni

**Affiliations:** ^1^Division of Geriatric Medicine, Department of Medicine, University of Verona, Verona, Italy; ^2^Division of Geriatric Medicine, Department of Surgery, Dentistry, Pediatric and Gynecology, University of Verona, Verona, Italy

**Keywords:** brown adipose tissue (BAT), beige adipose tissue, aging, inflammaging, metabolic disease

## Abstract

Across aging, adipose tissue (AT) changes its quantity and distribution: AT becomes dysfunctional with an increase in production of inflammatory peptides, a decline of those with anti-inflammatory activity and infiltration of macrophages. Adipose organ dysfunction may lead to age-related metabolic alterations. Aging is characterized by an increase in adiposity and a decline in brown adipose tissue (BAT) depots and activity, and UCP1 expression. There are many possible links to age-associated involution of BAT, including the loss of mitochondrial function, impairment of the sympathetic nervous system, age-induced alteration of brown adipogenic stem/progenitor cell function and changes in endocrine signals. Aging is also associated with a reduction in beige adipocyte formation. Beige adipocytes are known to differentiate from a sub-population of progenitors resident in white adipose tissue (WAT); a defective ability of progenitor cells to proliferate and differentiate has been hypothesized with aging. The loss of beige adipocytes with age may be caused by changes in trophic factors in the adipose tissue microenvironment, which regulate progenitor cell proliferation and differentiation. This review focuses on possible mechanisms involved in the reduction of BAT and beige activity with aging, along with possible targets for age-related metabolic disease therapy.

## Introduction

Aging is considered a common and a well-established risk factor for several chronic diseases, as well as for decline in physical function and frailty (1–3). Moreover, aging is associated with increasing prevalence in obesity, dyslipidaemia, type 2 diabetes, glucose intolerance and other comorbidities.

In recent years, the adipose organ has assumed considerable importance in aging and age-related metabolic dysfunction. Important and profound changes in the adipose organ occur with aging in terms of adipose tissue distribution and composition, and it has been suggested that progressive dysfunction of AT may represent an important hallmark of the aging process. AT dysfunction represents a process responsible for the metabolic alterations, the multi-organ damage and the systemic pro-inflammatory state (“inflammaging”) typical of aging itself (4). Data in the literature support the idea that adipose tissues are organized in a large adipose organ with discrete anatomy, vasculature and innervation, specific cytology and high plasticity (5). AT is distributed in several depots, localized into two main compartments: subcutaneous (SAT) and visceral (VAT) adipose tissue with different compositions and functions. The main cells of AT are represented by adipocytes, defined as white and brown adipocytes in relation to their different morphology, which reflects their different functions (5) (Table 1). Both types of cells are present within multiple sites of adipose organ in discrete depots and are named white and brown adipocytes.

**Table 1:** Main morphological and functional characteristics of white, brown, and beige adipocytes.

**Characteristics**	**White adipocytes**	**Brown adipocytes**	**Beige adipocytes**
Morphology	Spherical cells with a single cytoplasmatic lipid droplet and peripheral “squeezed” nucleus	Polygonal cells with several cytoplasmatic lipid droplets and a roundish nucleus	Paucilocular/Multilocular adipocytes with intermediate morphology
Ultrastructural morphology	Low mitochondrial content	Large, spherical and packed mitochondria which laminar cristae	High mitochondrial content
Innervation	Low noradrenergic fibers	Numerous noradrenergic fibers are found in fat lobules with blood vessels	-
Vascularization	5–7 times less vascularization than BAT	High vascularization	-
Markers	UCP-1 negative cells Leptin positive cells S100B positive cells	UCP-1 positive cells Leptin negative cells	UCP-1 positive cells S100 B positive cells Leptin positive
Localization	SAT and VAT depots and ectopic fat	Cervical-supraclavicular, perirenal and paravertebral regions and around the major vessels such as aorta	In various WAT human depots (inducible transition white to beige)
Embryological origin	WAT adipocyte precursors can derive from both *Myf5+* and *Myf5-* lineages	The same of skeletal muscle deriving from specific cells of the dermomiotome (from Myf5+ cells)	White-to-brown adipocyte transdifferentiation and *de novo* differentiation of precursor cells
Function	Storage of energy	Thermogenic activity	Thermogenic or storage phenotype depending on environmental conditions
Changes with aging:			
-Chronic sterile inflammation -Progenitor cell decline -Senescence -Adipokines changes	↑ ↑ ↑ ↑	↑ ↔ ↑ ↔	↑ ↔ ↑ ↔

*↑ = increased; ↔ = unchanged*.

At the morphological level, the main characteristic of white adipocytes is their single large intracellular lipid droplet (LD), while the brown adipocytes are characterized by the presence of multiple small cytoplasmic LDs (Table 1). White adipocytes have the function of storing excess lipids in the form of triglycerides (TG) and releasing free fatty acids (FFA) in periods of body energy demand; white adipocytes also synthesize and release adipokines which regulate metabolic homeostasis (Table 1). The main function of brown adipocytes is the dissipation of energy through uncoupled respiration so as to produce heat; this mechanism is mediated by a protein called the uncoupling protein-1 (UCP-1), present in the inner membrane of mitochondria (6) (Table 1).

A third type of adipocyte with an intermediate morphology between that of white and brown adipocytes, also referred to as beige, “brite” (brown-like-in-white) or “inducible brown” adipocytes, was firstly described in mouse WAT and then found in various human WAT depots (7, 8). Despite similarities to brown adipocytes, beige adipocytes can undergo a thermogenic or storage phenotype depending on environmental conditions (Table 1).

Across aging, AT undergoes changes in quantity and distribution, with an increase in total AT and VAT up to 65 years, as well as of ectopic fat deposition, and a decrease in SAT (9–11). Across aging, BAT declines, even if BAT activity may be identified in some rodents models and, under certain conditions, in human beings. However, the relevance and potential role of BAT decline with aging has still not been fully explored and determined.

## Brown Adipose Tissue: Anatomical Decline With Aging

The main deposits of brown adipose tissue in the mouse are located around the spinal cord in the paravertebral area and in the mediastinum (12), especially in the para-aortic area and around the heart, at the apex. Infradiaphragmatic deposits have also been described, in particular in the perirenal area, which occur in smaller quantities than the supra-diaphragmatic deposits (13).

Most of available information on BAT has been obtained from rodents because a carcass evaluation can be performed. In mice, white, brown, and mixed areas are present in discrete depots; some subcutaneous and visceral depots are clearly partitioned into WAT and BAT (14, 15). Under physiological conditions, the exposure of mice to cold increases the amount of BAT depots. After cold exposure, UCP-1-dependent pathways are also activated in subcutaneous WAT (sWAT), and in brite adipocytes through the activation of the sympathetic nervous system (16–18).

In adult humans there are functionally active areas of BAT, more frequently in women than in men, in particular in the cervico-supraclavicular region; however, the study of human BAT is difficult (19). In humans, the 18F-fluorodeoxyglucose (18F-FDG) positron emission tomography (PET-CT) computed tomography is the most common method for the measurement of metabolically active BAT, by the identification of AT regions that have a high assimilation of 18F-FDG on the PET scan. It is important to point out that the BAT detected using 18F-FDG PET-CT has been demonstrated to correspond histologically to BAT (20). [18F] FDG PET reveals glucose metabolism in tissues; in detail, active BAT of mice and humans preferentially combusts fatty acids derived from plasma triglycerides after lipolysis, which is fueled by glucose. Indeed, [18F] FDG PET does not detect directly metabolically active BAT, but it is still a reliable indicator of activated BAT (21, 22).

**Figure 1:**
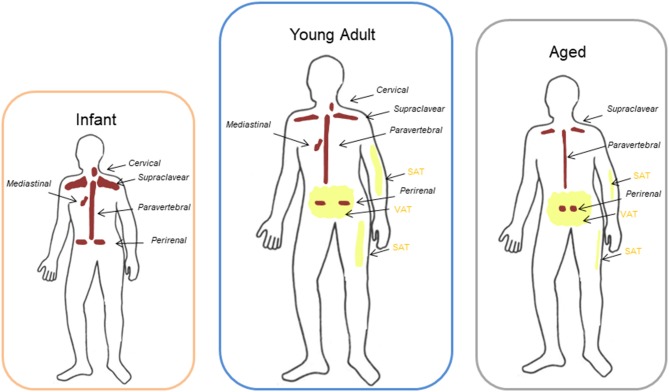
Different location of BAT and WAT at different ages. Brown adipose tissue (BAT) in infants and young adults had been described to be localized mainly in the cervical-supraclavicular region as well as in periaortic areas inside the thorax and the abdomen, and in particular in the perirenal fat. With aging the amount of detectable BAT decreases progressively and it remains represented mainly in the supraclavicular and perirenal sites. Peripheral depots (interscapular) are the first to loose BAT with increasing age, whereas deeper BAT depots, such as the perivascular or perirenal depots, decline in later stages of life. Aging is also characterized by a redistribution of white adipose tissue (WAT) with a progressive loss of subcutaneous adipose tissue (SAT) from limbs and an accumulation in trunk and abdomen of visceral adipose tissue (VAT) compared to adults.

In humans, BAT changes during the various stages of life (Figure 1). BAT begins to form during gestation, and it has a critical role in thermoregulation in the first phases of human life because newborns do not possess the ability to shiver yet. In infants, the large bilateral supraclavicular depot represents the most metabolically active form of BAT, which can be rapidly activated to heat production (Figure 1). During childhood, adolescent BAT is found mainly in the supraclavicular region but, although active BAT is present in every child, metabolically active BAT is detected only in about half of adolescents after cold exposure (23–25).

Moreover, other evidence suggests that there is an increase in BAT activity during adolescence, especially during sexual maturation and musculoskeletal development. Studies with FDG-PET/TC scans indicate that there is a synchronized growth of BAT and skeletal muscle during puberty and the development of these tissues is related (24, 25).

BAT function and mass decline with aging. Anatomical distribution of BAT is similar between adolescents and adults: most of the depots are located in the cervical-supraclavicular region and other depots are in axillary, mediastinal, paravertebral, epicardial and abdominal regions (26, 27) (Figure 1). Peripheral depots, such as interscapular one, are the first to lose BAT with increasing age, whereas deeper BAT depots, in particular perivascular or perikidney ones, decline in later stages of life (28–30) (Figure 1).

Non-stimulated BAT can be identified in people under the age of 50 years at a rate of three times more than in individuals older than 64 years old (26–31). Through the use of FDG-PET/TC to visualize BAT in living subjects, it has been shown that cold-stimulated BAT activity decreases with age (26, 27, 29). Loss of BAT may plateau around the sixth decade of life and then decrease in later years, and cold-stimulated BAT activity is rarely detected in individuals over the age of sixty; this could explain why there is a decrease in the ability of the elderly to tolerate cold temperature and to control body temperature (29). However, FDG PET/TC studies measure only activated BAT, which may or may not reflect changes in mass (29). The decline in BAT activity with age in humans is consistently supported by findings from rodents: in fact, the interscapular BAT depot in rats becomes infiltrated by white adipocytes with age, with an important decline in UCP1 activity and function (30). A significant loss of UCP1 with age is also observed in rats subcutaneous WAT, confirming the findings of human autopsy studies (31). It therefore seems that many, if not all forms of BAT, may undergo a gradual transition toward WAT with increasing age both in human as in rodent studies (30, 31).

In summary, aging is one of the most relevant determinants of BAT activity and it is associated with a ubiquitous decline of BAT activity throughout life (32, 33).

## Mechanisms Of Brown Adipose Tissue Decline With Aging

**Figure 2:**
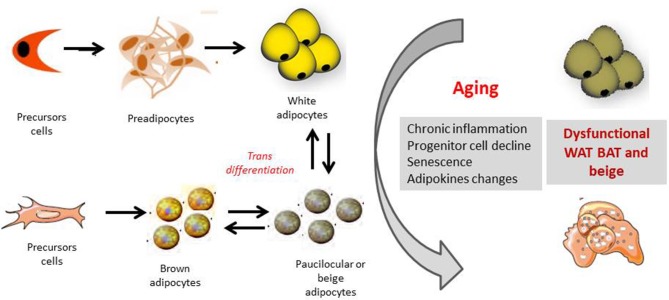
Orgin of white, brown and beige adipocytes and effect of aging. The appearance of beige cells among white adipose tissue (WAT) depots is referred to as “browning.” It has been hypothesized that these cells origin from the *de novo* differentiation of a distinct sub-population of WAT resident progenitors, which express the markers CD 137 and TMEM26 on their surface. Moreover beige-type cells may generate also from white-to-brown adipocyte transdifferentiation, from MYF5 positive cells. Several mechanisms as chronic inflammation, progenitor cell decline, senescence of the different adipose cell compartments as well as changes in adipokines productions, may together contribute to a dysfunctional adipose organ with aging.

Several mechanisms have been shown to be related to BAT decline with aging, including loss of mitochondrial function, impairment in the sympathetic nervous system and age-induced alterations in endocrine signals and inflammation (Figure 2).

### Impairments in Mitochondrial Activity

During aging, there is a significant decline in UCP1 activity, a protein uniquely expressed in the inner mitochondrial membrane of brown adipocytes. Mitochondrial dysfunction has been recognized to have a relevant role in the pathogenesis of several age-related disorders, such as type 2 diabetes, obesity, heart failure, neurodegenerative diseases and tumorigenesis (34, 35).

In particular, mitochondrial dysfunction with aging is characterized by an increase in mitochondrial DNA mutations and a reduction in biogenesis and oxidative phosphorylation. For this reason, aging may be associated with an impairment in brown adipogenic stem/progenitor cell function and consequently with a reduction in the regenerative potential of BAT with storage of dysfunctional brown adipocytes (34, 36).

### Decrease in the Sympathetic Nervous Stimulation and Sensitivity in BAT With Age

The sympathetic nervous system (SNS) plays a key role in regulation of BAT recruitment. Moreover, exposure to cold is a potent BAT stimulator through the SNS. Cathecolamines activate B3-adrenergic receptors located on the surface of brown adipocytes to promote UCP1 gene expression and activity related to thermogenesis and lipolysis (37, 38).

The sympathetic nervous system of BAT can be visualized by I-meta-iodobenzylguanidina SPECT (39). 123I-metaiodobenzylguanidine (123IMIBG), a radiolabeled norepinephrine analog, is commonly used for scintigraphic assessment of neuroendocrine tumors and cardiac sympathetic activity (39). 123I-MIBG scintigraphy, in particular, has already been used specifically to localize BAT in rats. Interestingly, a recent study in lean adult humans after cold exposure, measured BAT through the combination of the two imaging methods: the 123IMIBG SPECT/CT, as a measure of sympathetic stimulation and activation, and the 18F-FDG PET/CT, as an indicator of BAT metabolic activity (37–39). In older lean subjects, both sympathetic drive and BAT activity were lower compared to younger lean and obese men (37).

Therefore, it is possible that a lower absolute SNS signal and a possible decline in sensitivity of BAT for the SNS stimulation may result in a decreased ability to activate and recruit BAT in older men and may also explain why older humans have an inability to appropriately regulate their temperature when exposed to cold (37, 38).

### Age-Related Hormonal Changes and BAT

BAT mass and/or function may decline during adulthood, as a consequence of changes particularly in the somatotropic and gonadotropic axes.

Human fetal BAT shows a high expression of estrogen receptors, suggesting that tissue-levels of estrogens may regulate BAT activity (40, 41). Recent evidence suggests that estrogens and androgens are positively related to BAT activity and function while glucocorticoids have negative effects. Glucocorticoids, such as dexamethasone, reduce the cathecholamine-induced expression of UCP1 (42). With aging, sex hormone levels decline, while glucocorticoid levels remain relatively stable; for this reason, this decrement in levels of gonadotropic hormones in late adulthood and relative increase in glucocorticoid activity may contribute to the loss of BAT activity with aging (41, 42).

Thyroid hormones are also known regulators of thermogenesis: UCP1 levels are related to triiodothyronine levels. Extensive evidence shows that T3 promotes UCP1 synthesis at transcriptional levels in BAT, as well as UCP1 activity by modulation of cAMP production. Aging is associated with a decrease in serum T3 and a reduced conversion of active T3 caused by an age-dependent loss of DIO2, as is demonstrated in murine WAT (43). Recent evidences demonstrated that UCP1 expression in WAT also correlates with circulating thyroxine serum levels (43, 44), suggesting that thyroid hormones may contribute to the browning of white adipose tissue and increasing thermogenesis (45).

A few recent papers suggest ghrelin signaling as an important thermogenic regulator in aging. The ablation of the ghrelin receptor, the growth hormone secretagogue receptor (GHS-R), decreases the risk of age-associated obesity and insulin resistance. Ghrelin and obestatin are derived from the same preproghrelin gene; however, in brown adipose tissue, ghrelin reduces the expression of UCP-1 but obestatin increases it. During aging, plasma ghrelin and GHS-R expression in BAT are increased, but plasma obestatin is stable; this may lead to an imbalance in thermogenic regulation, which may in turn exacerbate age-related thermogenic impairment. Moreover, GHS-R ablation activates thermogenic signaling, enhances insulin activation, mitochondrial biogenesis, and improves BAT mitochondrial function (46, 47).

In recent papers, it has been suggested that fibroblast growth factor 21 (FGF21), secreted by the liver and adipose tissue, may also play a role in the browning process of WAT depots. In fact, mice with a FGF21 deficiency display an impaired ability to adapt to chronic cold exposure, with reduced browning of WAT. In particular, it has been demonstrated that adipose-derived FGF21 increases the expression of UCP1 and other thermogenic genes in fat tissues in an autocrine/paracrine manner (48, 49). However, aging is characterized by a progressive increase in FGF21 levels from 5 to 80 years, independently of body composition (49). This discrepancy between the increase in FGF21 levels and decrease browning of WAT with aging could be at least in part reconciled, hypothesizing an FGF21-resistant state with age itself. In fact, evidences from studies conducted on rodents supported the existence of an age-related FGF21-resistant state (50). Similarly, in some metabolic states such as obesity and diabetes, FGF21 levels are elevated and an FGF21-resistant condition has been suggested to also accompany these diseases (51).

### Inflammaging

The production of pro-inflammatory mediators and the infiltration of immune cells in AT, a process of chronic inflammation typical of obesity and metabolic conditions, also increases with age, so that has been referred to as “inflammaging” (52–54).

Several evidences have suggested that compared to WAT, brown and beige adipose tissue are less likely to undergo local inflammation due to immune cells infiltration, but present an increased production of inflammatory cytokines as TNF-alpha and MCP-1, in presence of dysregulated metabolism linked to obesity (53).

It has been hypothesized that inflammation with the production of these cytokines may indirectly impair the thermogenic activity in BAT because of altered insulin sensitivity and reduced glucose uptake (53).

Several pro-inflammatory cytokines are suggested to be involved in these mechanisms that globally reduce UCP-1 gene expression and browning phenomena (52, 54). In particular, TNF-a, one of the principal cytokines that increases during these inflammatory processes, via Toll-like receptor (TLR) activation induces apoptosis of brown adipocytes and inhibits the expression of UCP1 and b3-adrenergic receptor on adipocytes, ultimately decreasing thermogenesis in BAT (52). Other inflammatory mediators, such as pattern recognition receptors (PPR) and nucleotide-oligomerization domain containing proteins (NODs), have been suggested to have a critical role in modulating BAT activity during inflammation via activation of NF-kB and MAPK signaling pathways (53, 54).

## Beige Adipocytes And Aging

Beige/brite adipocytes are likely to originate from both a white-to-brown transdifferentiation mechanism and *de novo* differentiation from specific precursor cells (Figure 2).

The phenomenon by which the appearance of beige cells among WAT depots is observed is referred to as “browning” (55); this mechanism is mainly based on adrenergic signals and cold exposure. The beige-type cells in white fat depots are genetically different from those in classic interscapular and perirenal BAT, which are derived from myogenic factor 5 (MYF-5) positive precursors (56). Moreover, recent evidence suggests that both BAT and WAT adipocytes derive from the vascular endothelial cells of adipose tissue, supporting a role for transdifferentiation (57). This induction of beige adipocytes depends on several mechanisms, including, in addition to the aforementioned environmental temperature, genetics factors, diet, developmental periods and anatomic location of the adipose tissues (58).

With regard to the *de novo* differentiation, adipose stem and/or progenitor cells reside within WAT, which can proliferate and differentiate into either white or beige/brite adipocytes. In particular, a distinct sub-population of WAT resident progenitors, which express the markers CD 137 and TMEM26 on their surface, show a greater ability to differentiate into beige cells (59).

As a consequence of this, the age-related dysfunctional regeneration and reduction of classical brown and beige tissues could be due to a defective ability to proliferate and differentiate from inducible WAT or due to a loss of CD137/TMEM 26+ progenitors.

Moreover, different molecular mechanisms have been described that underlie the loss of beige adipose tissue during aging. Interestingly, SIRT1, an important target in AT biology (60), drives the browning of adipose tissue by promoting the interaction between PPARgamma and PRDM16, a potent inducer of beige adipose-specific genes (61). A recent study demonstrated another role of SIRT1, via the regulation of the senescence pathway p53/p21 (62). By reducing the expression of p53, a transcription factor of p21, SIRT1, enhances beige adipocyte differentiation capability of elderly adipose tissue-derived mesenchymal stem cells (AT-MSCs). During aging there is a reduction in SIRT1 levels, but how its expression is regulated into these stem cells is still unclear. Recently, it has been demonstrated the microRNA 34a (miRNA 34a) is a direct regulator of SIRT1 (63). Interestingly, miRNA-34a suppresses the process of browning under conditions of obesity, in part via its regulation of SIRT1 and FGF-21. This evidence supports its role of being a candidate molecule for improving the differentiation ability of elderly AT-MSCs as a treatment of aging obesity (63).

## Brown And Beige Aging: Potential Intervention Targets

White and brown AT has been suggested as a target for the prevention of type 2 diabetes, lipid disorders, as well as for delaying aging. Counteracting the age-associated loss of brown and beige adipose tissue could be an interesting and innovative therapeutic approach. Different types of strategies and molecular targets have been suggested for implementing possible interventions to slow age-related changes in brown and beige adipose tissue.

### Physical Exercise

Well known and widely studied are the effects of physical exercise on adipose tissue physiology, since it is well known that regular physical activity improves glucose tolerance and reduces white adipose tissue mass. Several studies have shown that physical exercise is associated with changes in both subcutaneous and visceral fat, a reduction of adipocyte size and lipid content, enhanced expression of metabolic pathway genes, modified secretion of adipokines and increased mitochondrial activity (64). However, less well-known and studied are the effects of physical exercise in AT of elderly humans, and in particular on BAT.

Studies evaluating the effect of exercise on BAT in old rodents and humans have been published with conflicting results. In a recent study conducted in old rats, both strength and aerobic training determined an increase in BAT, in mitochondrial activity, thus reducing total body fat (65). These results were also confirmed in other rodent models, where the physical exercise-associated browning of subcutaneous WAT has been demonstrated (66).

However, these findings were not confirmed in elderly subjects as a decrease in mitochondrial activity and in glucose uptake in BAT after training has been shown (67).

More examination is mandatory to completely understand the impact of the complex relationships between different types of exercise and exercise-induced adaptations in WAT and BAT, as it is a field of study of considerable interest.

### Nutritional Strategies

Since intermittent fasting was proved to optimize energy metabolism (68), in a recent study rodents were kept on an every-other-day fasting regimen: this approach favored the activation of beige fat thermogenesis and improved obesity-related metabolic diseases, probably via a microbiota-beige fat axis (69). Interestingly, in a human model, Orava and colleagues observed that insulin stimulated a 5-fold increase in FDG uptake in BAT, suggesting that BAT may contribute to postprandial energy metabolism in humans (70).

Recent researches have demonstrated that some food ingredients may be involved in promoting energy expenditure and fat oxidation in BAT. In fact, it has been shown that capsaicin and its analogs in hot peppers, as well as caffeine and catechins in green tea, could be related to an increased energy expenditure (71, 72). In particular, capsaicin may induce a browning program in WAT, stimulating UCP-1 and promoting SIRT1 expression and activation through TRPV1 (transient receptor potential channels) channels (73). Josse et al. have reported that an ingestion of meals supplemented with capsinoids may increase energy expenditure and lipid oxidation through an activity on Beta3-adrenergic receptors (74, 75).

It has also been shown that PUFAs (Long-chain omega-3 polyunsaturated fatty acid) and, in particular, eicosapentaenoic acid (EPA), known for their anti-inflammatory and cardio protective effects, reduce high-caloric diet related obesity and insulin resistance in mice. The mechanism underlying BAT activation seems to depend on FGF21 expression, also without cold exposure, suggesting that EPA supplementation alone may mimic the cold induced BAT activation (76).

All of this evidence may demonstrate future area of research for a non-pharmacological strategy to treat obesity and age-related metabolic disease, also in elderly individuals.

### Cold Exposure

It is now widely known from studies on mice and humans that exposure to cold increases the activity of brown adipose tissue (27). Saito et al. found an unexpected high presence of cold-activated BAT by performing FDG-PET/CT scans in healthy adults under warm and cold conditions, which suggests an important role of temperature in the regulation of BAT activity and body fat content. More recently, gene expression profiles and metabolic pathways activated in BAT exposed to cold have been investigated; in an interesting study conducted in mice, it was shown that cold exposure highly influenced BAT metabolic activity. Exposure to cold is characterized by lower levels of glycolysis and gluconeogenesis intermediates, higher levels of tricarboxylic acid cycle metabolites, free fatty acids and acyl-carnitine metabolites, suggesting that glycolysis and β-oxidation of fatty acids in BAT are biological pathways that contribute to increased thermogenesis by cold exposure (77, 78).

Recent studies have evidenced that mitochondrial reactive oxygen species (ROS) play an important role in modulating thermogenesis and UCP1 activity. In fact, cold exposure is characterized by an increased mitochondrial superoxide and oxidation of lipids and proteins in BAT. Furthermore, acute cold exposure leads to enhanced oxidation and a decrease of reduced glutathione in BAT; this process is associated by increased protein thiol oxidation, which has been suggested as a vital signaling mechanism required for UCP1-induced thermogenic metabolism (79, 80).

### Pharmacological Strategies

The PPARgamma agonist rosiglitazone exerts its thermogenic effects on adipocytes by increasing PRDM16 (regulator PR domain containing 16) protein half-life, a zinc-finger transcriptional factor that plays a key role in the differentiation of adipocytes (61). In fact, PRDM16 controls the bidirectional switch between brown adipocytes and myoblasts. PRDM16 determines the brown fat-like gene expression and thermogenesis in both BAT and WAT. Moreover, the expression of this transcriptional regulator is strongly correlated with beige cell-selective genes, in the so-called browning process (61). From a therapeutical point of view, some reports have supported extensive inhibition of adipokines production, including resistin, 1-acidglycoprotein, and haptoglobin, by treatment of white adipocytes with thiazolidinedione (TZD) and non-TZD synthetic PPARgamma ligands (81).

Moreover, Finlin et al. have suggested that mirabegron, a beta 3-agonist, induces the expression of UCP1 and beige adipocyte markers to a higher degree than 10 days of repeated cold exposure. This phenomenon may be exploited to increase beige adipose tissue in older, insulin-resistant, obese individuals (82).

Vitamin A metabolites or retinoids are other hormones required for BAT activation. Retinoic acid strongly induces UCP1 expression in adipocytes, indicating that body thermogenic capacity may also be related to the vitamin A status. Retinoic acid may also determine adipocyte differentiation and survival, with high doses inhibiting and low doses promoting adipogenesis of adipose cells precursors *in vitro* (83). The administration of retinoids in high-fat diet mice is associated with an increase in adipose UCP1 expression and a reduction in body weight. However, its role in humans is still controversial: UCP1 expression is differently affected by all-trans retinoic acid (ATRA) in mouse and human adipocytes (84).

Bone morphogenetic proteins (BMPs) are a family of secreted molecules that contribute to the differentiation of mesenchymal stem cells and drive the formation and thermogenic activation of BAT (85). BMP9 treatment has been shown to determine the browning of subcutaneous WAT, to improve glucose tolerance and reduce weight gain in *in vivo* experiments (86). A role for BMP8B in the regulation of thermogenesis has also been described; this protein regulates nutritional and thermogenic factors in mature BAT, improving the response to noradrenaline through enhanced p38MAPK/CREB signaling and increased lipase activity. *Bmp8*b^−/−^ mice show impaired thermogenesis and decreased metabolic rate, determining weight gain despite hypophagia. BMP8B is also expressed in the hypothalamus, and *Bmp8*b^−/−^ mice display altered neuropeptide levels and reduced phosphorylation of AMP-activated protein kinase (AMPK), indicating an anorexigenic state (87).

Fibrates exert their lipid-lowering activity via PPARalpha. The new compound, GW9578, was demonstrated to enhance insulin sensitivity and to decrease adiposity *in vivo* (88). Moreover, the PPARalpha agonist GW9578 stimulates expression of the thermogenic gene program in beige adipocytes and rescues the beige-to-white fat transition phenotype induced by loss of Lsd1 (89).

However, most of the research about transcriptional factors involved in the regulation of BAT development has been carried out in mice and the expression of these marker genes seems less consistent in the humans, with a lack of specific data for elderly subjects in particular.

## Conclusions

Further human studies are needed to investigate the effectiveness of nutritional and pharmacological stimulation to maintain BAT and beige mass and sensitivity during aging.

However, any therapeutical targeting of BAT activity and/or mass will first require a clear understanding of the mechanisms involved in potentiating BAT activity.

## Author Contributions

All authors listed have made a substantial, direct and intellectual contribution to the work, and approved it for publication.

### Conflict of Interest Statement

The authors declare that the research was conducted in the absence of any commercial or financial relationships that could be construed as a potential conflict of interest.

## Abbreviations

BAT: brown adipose tissue
WAT: white adipose tissue
SAT: subcutaneous adipose tissue
VAT: visceral adipose tissue.
